# Syndactyly in the Pediatric Population: A Review of the Literature

**DOI:** 10.7759/cureus.36118

**Published:** 2023-03-14

**Authors:** Sonal Mahindroo, Sean Tabaie

**Affiliations:** 1 Orthopedic Surgery, George Washington University School of Medicine and Health Sciences, Washington DC, USA; 2 Orthopedic Surgery, Children's National Hospital, Washington DC, USA

**Keywords:** deformities, congenital, hand, pediatrics, syndactyly

## Abstract

Syndactyly is one of the most common congenital upper extremity deformities. Syndactyly can be described as either simple, involving just the skin and soft tissue, or complex, involving the phalanges. Additionally, syndactyly can be categorized as complete, involving the entire digit (including the nail fold), or incomplete, which does not involve the nail fold. Multiple familial or spontaneous genetic abnormalities can cause syndactyly, and these mutations typically involve the canonical wingless-type (WNT) pathway. Surgical repair of syndactyly is typically done between six to 18 months of age, depending on the type of syndactyly. Regardless of the classification of the syndactyly, the repair is performed before school-going age (except in the case of extremely mild or rare, extremely complex syndactyly).

One or more imaging modalities are used to aid the surgeon in deciding the surgical approach for the syndactyly repair. The surgical plan must be clearly communicated with parents to manage expectations of aesthetics and function of the digits post-surgery. In brief, a syndactyly release surgery involves the creation of the web space using a geometrical design of the surgeon’s choice, defatting of finger flaps, separation of the digits, and closure with absorbable sutures. However, the approach may vary depending on the patient.

A “best” approach for rectifying the difference in surface area of separated versus fused digits has not yet been determined. While this was typically done using a skin graft, the use of alternative methods (most notably, using a synthetic dermal substitute or not using a graft at all and allowing the skin to heal with secondary intention) has been on the rise given the undesirable side effects of a graft. Less commonly, an external fixator can be used to expand soft tissue and skin. In the case of complete syndactyly, the Buck-Gramcko technique is most commonly used for nail flap reconstruction. Complications of the surgery include contracture, web creep, and the need for a second surgery. Thus, parents must be counseled in recognizing signs of complications.

## Introduction and background

Syndactyly is a congenital condition that involves the fusion of either one or multiple adjacent digits [1]. Syndactyly is the most common congenital hand deformity, representing about 20% of them, and occurring in about one in every 2000 to 3000 births [2-4]. It is seen to be twice as common in males than in females and more common in those of Caucasian descent [4]. Polydactyly is the second most common congenital hand deformity; many cases of polydactyly will present concomitantly with syndactyly [2]. Syndactyly interferes with normal hand function and surgery is typically required to regain function. About half of all cases are bilateral and more than half involve syndactyly of the third web space (between the middle and ring finger). However, the presentation of syndactyly can be very variable and a classification system is used to categorize and thus guide the treatment of the syndactyly [1]. The presentation of syndactyly can be heterogeneous even between the limbs of the patient [2]. Some cases of syndactyly are much less severe, only involving minor webbing due to incomplete penetrance, and thus may not require surgery at all [3]. The predictability of certain acute and complex complications when treating syndactyly requires discussion with the parents of the patient before the surgery takes place as well as follow-up visits for the patient for many years, sometimes into adulthood depending on the severity [1].

## Review

Types of syndactyly

**Figure 1:**
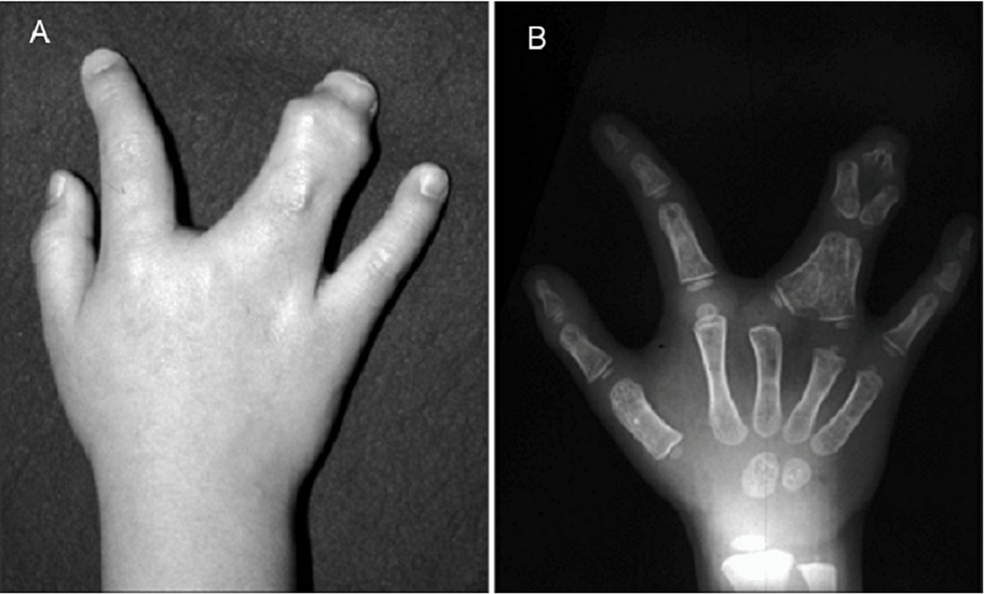
(A) Right-hand syndactyly between the distal and proximal, but not the intermediate, phalanges; (B) Radiograph of right-hand syndactyly Open access images courtesy of Ogino et al. [9]

Simple syndactyly occurs when there is a fusion of the skin and soft tissue, while complex syndactyly also involves the fusion of adjacent phalanges [5]. About 16.5% of cases of syndactyly are complex, and females are more likely to have complex cases [5,6]. Fusion of adjacent digits can either be complete, where the entire digit including the nail fold has fused or incomplete, where there is no nail fold involvement [7]. Polydactyly or the presence of extra digits can also be seen in some cases of syndactyly (termed synpolydactyly). Syndactyly may present either in it or by itself (isolated) or as part of a syndrome (syndromic) [8]. An example of a complete complex syndactyly is shown in Figure 1.

Common conditions associated with syndactyly include Poland and Apert’s syndrome, although syndactyly is a feature of over 300 different syndromes [3]. Isolated syndactyly (which is more common than syndromic syndactyly) usually involves syndactyly of the third or fourth web spaces, while syndromic syndactyly usually involves syndactyly of the first or second web spaces [10]. As cases of complex syndactyly are typically syndromic, a full clinical examination must be done to determine the presence of associated syndromes. Clinical judgment must be used before performing a full clinical examination on patients with simple syndactyly as they are not as likely to be associated with a syndrome [11]. Complicated syndactyly, which presents with additional bony abnormalities, is also indicative of an underlying syndrome [12].

Embryology

Most cases of syndactyly have a genetic basis, with 10% to 40% of cases presenting with a family history of syndactyly [11]. Hand development usually begins on day 27 of fetal development, which is around the time when other organs are also developing; this explains the occurrence of syndromic cases [12]. Syndactyly occurs when there is a defect in apoptosis of cells that make up the web space around week six of development, which is when the web space would usually regress [11,12]. Defects typically involve the canonical wingless-type (WNT) pathway, but other pathways have also been implicated in this process [13]. Apoptosis of the web space occurs in a distal to proximal manner, which explains the high occurrence of simple incomplete syndactyly [11]. The inheritance pattern of these genetic abnormalities is autosomal dominant (although they can also occur spontaneously), and once inherited, can have a variable expression or reduced penetrance. This explains why different generations may have vastly different presentations and severities of syndactyly [10]. Hypotheses of acquiring spontaneous genetic mutations include maternal smoking, poor maternal nutrition, and/or consuming excessive amounts of meat and eggs during pregnancy, low socioeconomic status, and mothers 40 years or older [10,13].

Timing of surgery

Simple syndactyly is usually repaired between 12 to 18 months of age, while complex syndactyly or simple syndactyly where fused digits have a significant difference in length should be repaired before six months of age [6,9,14,15]. If fused digits with a difference in length are not repaired in a timely fashion, this may cause a functional deformity due to asymmetrical growth [11,15]. An important consideration for deciding when to perform surgery on patients with syndactyly is determining when the patient will begin attending school. Ensuring that the patient has the proper function of digits (including the ability to grip and pinch) before school age is important for the development of not only their fine motor skills but also for psychosocial development; thus, all syndactyly release should be performed preferably before 24 months [16]. While surgery should not be delayed due to the risk of joint deviation, contracture, and web creep, performing surgery when the patient is too young increases the risk of anesthesia and scar contracture [4,15]. If syndactyly is bilateral, the surgery can occur at the same time [11]. However, if one digit is involved in two fusions, the surgery should not be done at the same time due to the risk of neurovascular compromise in the flaps and digits [3]. In this case, the surgeon should wait at least three months before the next surgery. If all digits are fused, which may be the case in patients with Apert’s syndrome, the first surgery should separate the first and third web spaces, and the second surgery should separate the second and fourth web spaces [4,10].

Surgery indications

Syndactyly is a clinical diagnosis; however, a radiograph must be performed for not only confirmation but also to determine the presence of hidden polydactyly or other abnormalities within the web space. Magnetic resonance imaging or ultrasound may also be indicated to determine tendon and/or muscle involvement to decide the best course of action for surgery [10]. A hand fellowship-trained plastic or orthopedic surgeon typically performs the surgery [6]. The surgeon performing the surgery must communicate to parents about the various facets of the surgery. The surgical strategy (including the use, or lack, of a graft) should be communicated to the parents, as well as postoperative care, and the potential need for corrective surgery as the patient grows (if the expansion of the skin graft is not congruous with bone growth). The surgeon must also emphasize that scarring may occur, and that function rather than aesthetics is the number one goal of syndactyly release surgery [11]. The surgery is typically performed in an outpatient setting, although very complex or complicated cases of syndactyly may be done in an inpatient setting at the surgeon’s discretion [6].

Contraindications for surgery

Almost all cases of syndactyly indicate that surgery should be performed. Some contraindications include extremely mild cases of syndactyly where the function is not impaired or certain cases of extremely complex or complicated syndactyly [10]. This could be due to a high risk of contracture that would impair mobility, a digit missing important elements that would further impair function if separation occurs, or if the patient has a medical condition where surgery, in general, is not indicated [16].

Surgical techniques

**Figure 2:**
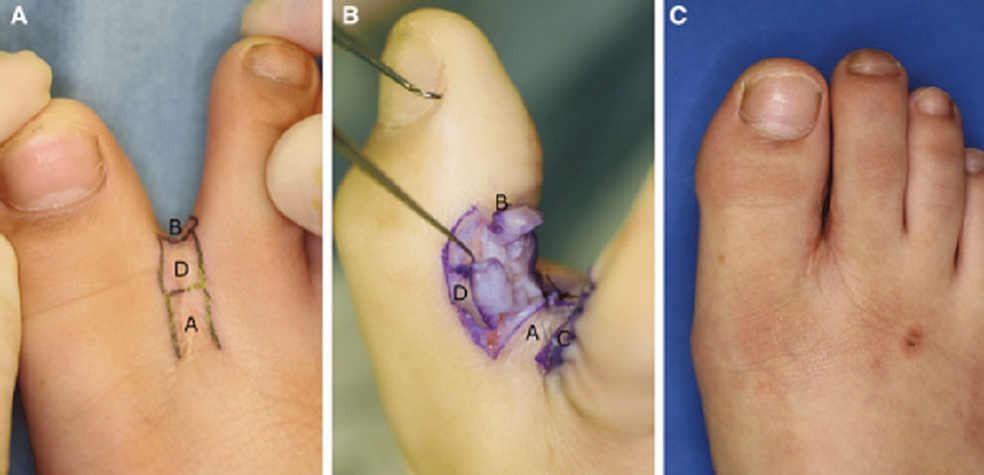
Syndactyly release in the fusion areas distal to the proximal interphalangeal joint A: An illustration of the three-square flap method, B: Separation of the digits, C: Postoperative follow-up after two years, showing no necrosis or other complications. Open access images courtesy Naoshige et al. [18]

Various techniques are used in syndactyly release depending on the nature of the syndactyly. The most common techniques are outlined below. A dorsal flap is typically used in the creation of the web space. Several geometrical designs can be used for the dorsal flap, including but not limited to rectangular, triangular, multilobed, and many others also being used [9,17]. An example of a syndactyly release using square flaps is shown in Figure 2.

The sides of the digits are usually separated using zig-zag folds. Whichever technique is used, it must be marked on the dorsal side, with a mirror image marking on the volar side. Finger flaps are defatted prior to closure to prevent suture tightening and ischemia. Great care must be used to take note of the neurovascular bundles while making incisions to ensure that they are well protected [19]. Fingers should be separated from distal to proximal. It is important to know that the bifurcation of the digital artery limits the location of the new webspace, and is the location that must be noticed as the surgeon is separating more and more proximally. Fine, absorbable sutures are used for closure, which will be done from proximal to distal. This prevents the need for skin grafts in this area [17].

Skin Flap with Graft

The surface area of separated digits is often much greater than that of fused digits. A graft must be used if the surface area is greater than 22%, as skin alone will not be able to rectify this difference [3,9]. This is especially the case in complete syndactyly [15]. Typically, grafts are taken from the groin area, lateral to the femoral artery (closer to the anterior superior iliac spine). However, this area seems to have a high incidence of hair growth and discoloration as the patient reaches the pubescent stage. Grafts from the volar wrist area in particular have shown to be successful, as there is a decreased incidence of hair growth. Additionally, the volar creases disguise scars well [20]. Grafts from the medial upper arm or cubital crease could also be used, although this may not be indicated in patients of all ages [10]. Full-thickness grafts are preferred to split-thickness grafts due to decreased incidence of contracture (although split-thickness grafts do have the benefit of being hairless) [15]. Synthetic dermal substitutes could also be used in place of a graft [21]. An initial study using MatriDerm (MedSkin Solutions, Billerbeck, Germany) required an additional graft, while the use of Integra (Integra LifeSciences, Plainsboro, NJ, USA) and Hyalomatrix (Anika Therapeutics Inc., Bedford, MA, USA) have been successful with no need of additional grafts [22-24]. Early studies indicate that using dermal substitutes results in minimal web creep and scarring and decreased operative time, although more studies are needed to determine the long-term effects of using a dermal substitute [25]. Complications of using a graft include hyperpigmentation, hair growth, graft failure, web creep, decreased range of motion, and keloid formation [15,26].

Skin Flap with No Graft

In the last two decades, there has been a shift towards avoiding the use of a skin graft, especially due to the inconsistent growth rate of the transplanted skin causing scarring and contracture. This method is indicated if the syndactyly is simple, incomplete, and does not extend past the proximal interphalangeal joint [7]. To overcome the issue of the surface area of the separated fingers being too great for the skin to rectify, extensive defatting must occur. However, too much defatting may lead to neurovascular damage, issues with venous drainage, and the fingers appearing emaciated [10,15,27]. Using a skin flap without the graft leads to less operative time, better skin match, and less scarring, leading to greater patient satisfaction. However, a rigorous risk versus benefits analysis must be done in order to determine if the patient is a good candidate for no grafting. In areas that are less than 2 mm in size, they can be left to heal with secondary intention, although there is a risk of increased scarring the larger the area is [10].

External Fixator

While not well studied, there have been instances of surgeons using an external fixator, especially in patients with simple complete syndactyly. Once Kirschner wires are added from the tip to the base of each fused digit, an external fixator is applied [7]. It is pulled slightly at every set increment of time to expand soft tissue and stretch the skin, ultimately preventing the need for skin grafts. This technique preserves blood supply to the area while being minimally traumatic, thereby resulting in better healing with reduced incidence of infections [28]. Early studies show that using an external fixator is successful at preventing postoperative complications, although it is also seen to increase the risk of rotational deformities and stretching of the nail [15].

Nail Flap and Fingertip Reconstruction

The Buck-Gramcko technique is used when there is nail involvement, as would be the case in complex syndactyly. A dorsal incision is made on the nail, and lateral digital flaps are made on the distal pulp [20]. The flaps are taken from the distal pulp and will help reconstruct either side of the digit [4,13]. Some complications of using the Buck-Gramcko technique include a lower aesthetic outcome (due to a bulky-looking fingertip), and in some cases (especially those that were syndromic), necrosis. So, a traditional zig-zag incision and full-thickness skin graft are indicated in patients with complete simple syndactyly [29].

Post-surgical care

Postoperative dressings are imperative to provide compression along the grafted areas and also between digits to allow for separation. In particular, moist cotton is placed between the newly separated digits, after which the extremity is reinforced with gauze. In younger patients, it may be indicated to reinforce the gauze applied after surgery with a plaster of Paris (POP) cast [30]. The POP will extend above the elbow and help reduce the mobility of the digits and will also prevent dressing slippage [30]. The dressing will remain on for about two weeks, depending on healing, and parents must be educated on the recognition of postoperative complications such as superficial site infections. After dressings are removed, gentle washing is done at the area of surgery, and normal use of digits is encouraged. Typically, formal physical therapy is not needed [10].

Surgery complications

Most surgical complications are associated with cases of complex or complicated syndactyly. These cases have a higher incidence of postoperative ventilator dependence, contracture, web creep, and hematomas [10]. Simple syndactyly surgeries are not without risk: 10% of cases will need secondary surgery [11]. Skin necrosis, due to nerve or arterial injury resulting in decreased vascularization of the skin flap is a major cause for concern during surgery. Infection, flap maceration, and graft rejection are worries in the post-operative time frame. Web creep will commonly occur if the surgery is done before 18 months or if split skin grafts have been used; this can be prevented by over-compensating the webspace by deepening it more proximally than usual [2].

## Conclusions

Syndactyly is a common congenital hand deformity that can manifest in an isolated or syndromic form. Patients can present with simple syndactyly, which occurs when the web space moves distally and there is a fusion of the soft tissue and skin but not of the nail bed, or with complex syndactyly, where there is bone involvement in addition to skin and soft tissue involvement. Patients can also present with either complete syndactyly, which occurs when there is the involvement of the entire digit, or with incomplete syndactyly, where only a portion of the digit is involved. Generally, syndactyly release will occur during school-going age (and typically between the ages of 12 to 18 months). There should be several separate surgeries if one digit is involved in multiple fusions, as performing both releases in one surgery can lead to neurovascular compromise. Several techniques have been used to separate the syndactylous digits, including the use of the graft, not using a graft, using synthetic dermal substitutes, or application of an external fixator to stretch the skin and soft tissue. Gauze will be placed between the digits post-surgery, which may or may not be reinforced with a POP cast. Complications such as contraction, hematomas, and necrosis may occur after the syndactyly release. If complications such as web creep occur after surgery, another surgery may be indicated. The surgeon must maintain strong communication with parents to explain the surgical plan, postoperative care, and signs that complications are occurring.
